# Full-Genome Sequence of Bluetongue Virus Serotype 1 (BTV-1) Strain Y863, the First BTV-1 Isolate of Eastern Origin Found in China

**DOI:** 10.1128/genomeA.00403-13

**Published:** 2013-07-11

**Authors:** Jianbo Zhu, Heng Yang, Huachun Li, Lei Xiao, Jinpin Wang, Nan Li, Nianzu Zhang

**Affiliations:** Yunnan Tropical and Subtropical Animal Viral Disease Laboratory, Yunnan Animal Science and Veterinary Institute, Yunnan, People’s Republic of China

## Abstract

The full-genome sequence of the bluetongue virus serotype 1 (BTV-1) strain Y863, the first BTV-1 isolate of Eastern origin found in China, was determined. The virus was isolated from sheep during a severe outbreak of bluetongue in Shizhong County, Yunnan Province, southwest China, in 1979. The total size of the BTV-1 strain Y863 genome is 19,170 bp. Phylogenetic analyses demonstrate that it belongs to the major “Eastern” BTV topotype. The sequence information provided here will help in understanding the geographical origin and spread of this Chinese isolate of BTV-1, as well as aid in its comparison with global isolates of BTV-1 from sheep, cattle, and other host species origins.

## GENOME ANNOUNCEMENT

*Bluetongue virus* (BTV) is the type species of the genus *Orbivirus*, within the family *Reoviridae* (1). The genome of BTV consists of 10 linear double-stranded RNA segments encoding the seven structural proteins (VP1 to VP7) and four nonstructural proteins (NS1, NS2, NS3/NS3a, and NS4) (2, 3). To date, twenty-six BTV serotypes (4, 5) have been described worldwide, and seven BTV serotypes (BTV-1, BTV-2, BTV-3, BTV-4, BTV-12, BTV-15, and BTV-16) have been isolated in China (6–9). BTV-1 and BTV-16 are the two most prevalent serotypes in China, and each serotype causes a disease with obvious clinical signs in infected sheep.

BTV strains show variations in their nucleotide sequences that reflect their origins from different geographic regions around the world (10), with a clear division of most genome segments into “Eastern” and “Western” groups or topotypes (11, 12). Geographical separation over long periods of time has allowed bluetongue viruses in different regions to acquire unique point mutations, some of which may make them particularly well suited to transmission and survival in their local ecosystems (10). Although full-genome sequences are available for one BTV-1 isolate, SZ97/1, from China, the sequence of SZ97/1 has high similarity with a South Africa BTV-1 strain and obviously is of “Western” origin (13). It was therefore important to characterize the complete genome of a native BTV-1 reference strain from China for molecular epidemiology studies.

We report here the complete genome sequence of a BTV-1 strain, Y863, isolated from sheep with severe bluetongue clinical signs during an outbreak that was first recognized in China in Shizhong County, Yunnan Province, in 1979 (14). Viral double-stranded RNA (dsRNA) preparation, full-length cDNA synthesis, PCR amplification, and sequencing of Y863 were performed according to the protocol described by Yang et al. (15).

The sizes (in base pairs) of Y863 segments 1 to 10 are 3,944, 2,940, 2,772, 1,981, 1,763, 1,635, 1,154, 1,125, 1,052, and 804, respectively. They encode proteins with amino acid lengths as follows: VP1, 1,302; VP2, 961; VP3, 902; VP4, 644; VP5, 526; VP6/NS4, 330/77; VP7, 349; NS1, 552; NS2, 354; and NS3/NS3a, 229/216. Further phylogenetic sequence analyses show that Y863 groups within the major “Eastern” BTV topotype. The data presented here report the complete sequence of a native BTV-1 isolated in China that belongs to the Eastern group. It will facilitate future investigations of the molecular characteristics and geographic origins of BTV-1 strains from China, as well as from other countries.

### Nucleotide sequence accession numbers.

The full-genome sequence of the BTV-1 strain Y863 was deposited in GenBank under the accession no. KC879615 to KC879624, corresponding to Y863 segments 1 through 10.
